# Gender Differences of B Cell Signature in Healthy Subjects Underlie Disparities in Incidence and Course of SLE Related to Estrogen

**DOI:** 10.1155/2014/814598

**Published:** 2014-02-13

**Authors:** Hongye Fan, Guanjun Dong, Guangfeng Zhao, Fei Liu, Genghong Yao, Yichao Zhu, Yayi Hou

**Affiliations:** ^1^The State Key Laboratory of Pharmaceutical Biotechnology, Division of Immunology, Medical School, Nanjing University, Nanjing 210093, China; ^2^Department of Physiology, Nanjing Medical University, Nanjing 210029, China; ^3^Jiangsu Key Laboratory of Molecular Medicine, Nanjing 210093, China

## Abstract

The aim of the present study was to investigate mechanism of the gender differences of B cells. The results showed that 358 differential gene expressions (DEGs) were displayed between healthy females and males. Compared with male, 226 and 132 genes were found to be up- and downregulated in the female. 116 genes displayed possible correlation with estrogen. Moreover, the upregulated DEGs (Cav1, CD200R1, TNFRSF17, and CXCR3) and downregulated DEGs (EIF1AY and DDX3Y) in healthy female may be involved in gender predominance of some immune diseases. Furthermore, signaling pathway analysis for estrogen-relevant DEGs showed that only 26 genes were downregulated in SLE female versus SLE male, of which expressions of 8 genes had significant difference between SLE females and SLE males but are having nonsignificant difference between healthy females and healthy males. Except for the 5 Y-chromosome-related genes or varients, only 3 DEGs (LTF, CAMP, and DEFA4) were selected and qRT-PCR confirmed that the expressions of LTF and CAMP decreased significantly in B cells from female SLE patients. These data indicated that the gender differences were existent in global gene expression of B cells and the difference may be related to estrogen.

## 1. Introduction

Gender differences are universal in health and the onset and the progression of the disease as well as in medical and pharmacological research. Clinical and basic science investigations have shown important sex differences in the development of neural systems [1], cardiac structure and function [2], risk of cancer [3], renal disease [4], and mental disorder [5]. The differentially expressed genes (DEGs) revealed sex-related differences in mRNA expression of kidney. The signaling pathway analysis of these sex-different genes indicated overrepresentation of specific pathways and networks [4, 6].

Clinical and experimental researches also have demonstrated naturally occurring gender differences in immune response [7, 8] and the distribution of lymphocyte subsets [9]. Women had higher levels of CD4+ and CD8+ T cell activation and inflammation-associated gene expression [10]. Lymphocyte subset enumeration revealed higher B cells in females [11]. Females are protected against mortality arising from severe sepsis, which may be attributed to a fundamental sex difference in phenotype of resident leukocytes [12]. Some studies on healthy humans considered the gender differences in immunosenescence [13–15]. Interestingly, some lines of evidence showed that cells of males and females also may display several different features and behaviours [16, 17]. These data suggest that the gender-dependent differences may have a biological base and molecular mechanisms. However, although it has been well known that immune responses are sexually dimorphic [10], specific genes responsible for the relationship between gender differences of immunity remain poorly understood.

B cells belong to a group of white blood cells known as lymphocytes, making them a vital part of the immune system. The principal functions of B cells are to make antibodies against antigens, to perform the role of antigen-presenting cells (APCs), and to develop into memory B cells after activation by antigen interaction. Moreover, as is well known, systemic lupus erythematosus (SLE) is the prototypic systemic autoimmune disease. Several lines of evidence support roles of B cells and autoantibodies in lupus pathogenesis in mice as well as humans [18]. Loss of B cell tolerance is a hallmark feature of the pathogenesis in SLE and abnormal B cell homeostasis has been described in SLE patients [19]. B cells from SLE patients are hyperresponsive to a variety of stimuli [20]. Furthermore, the findings in mouse models suggest that defective regulation of intracellular signaling in B cells could directly lead to lupus-like autoimmunity [21]. The B cell depletion with rituximab improves the clinical manifestations of SLE [22]. Importantly, SLE also possesses a gender-dependent incidence characterized by a male/female ratio of 1 : 9. Thus, it is necessary to elucidate the biological basis of B cells in gender differences for understanding the onset and the progression of SLE.

Microarray analysis is a comprehensive profiling method and has been used by many investigators to study differential gene expression. The early studies mainly used total peripheral blood mononuclear cells, and as a consequence the majority of the disease-related signatures identified simply reflected differences between patients and controls. Moreover, the peripheral B cell transcriptomes of quiescent lupus patients were compared to normal B cell transcriptomes. This microarray analysis of B cells during quiescent lupus suggests that, despite a similar lupus phenotype, different biological roads can lead to human lupus [23]. Recently, Becker et al. have reported active SLE peripheral blood B cell transcriptome profiles and found that the elevated expression of a number of transcripts was related to cytokine activation, cell differentiation, cell cycle regulation, and apoptosis [24], which are similar to our results in active SLE B cell signature. However, very few information has been acquired so far as concerns gender differences of the B cells for understanding the female predominance.

In addition, substantial studies suggest that gender differences may be controlled by different levels of gonadal steroid hormones [25]. It is generally recognized that the male hormone, testosterone, was immunosuppressive, while the female hormone, estrogen, stimulated immune response [25, 26]. These hormones changed the distribution and function of lymphocyte subsets [26, 27]. Our previous studies also showed that 17*β*-estradiol upregulated the proliferation and function of lymphocytes [28–31]. Moreover, besides genetic effects, possible reasons for the differing incidence and prevalence of autoimmune diseases may be related to sex hormones [32]. However, there is limited knowledge concerning the molecular mechanism of lymphocytes in gender differences between immune function in healthy subjects and the incidence of some immune diseases.

Therefore, the comprehension of mechanisms of the gender differences of B cells may disclose new scenarios not only for the cell physiology and pathology per se, but also in preclinical and clinical management of some autoimmune diseases including SLE. In the present study, gene expression signature of B cells in sex differences was undertaken for understanding the female predominance of SLE. Bioinformatics analysis of the microarray results was performed. In addition, mouse B cells were obtained to verify whether the change of some gene expression is related to estrogen. Results showed that the gender differences were existent in global gene expression of B cells. This difference may be related to estrogen. These findings suggest that the diagnosis and treatment of these immune-related diseases should consider the baseline gender-related differences.

## 2. Materials and Methods 

### 2.1. Patients and Healthy Subjects

The study protocol was approved by the Research Ethics Committee of Nanjing University. After obtaining written informed consent from each SLE patient and healthy subject, peripheral blood samples were obtained from SLE patients and healthy subjects. All SLE patients were diagnosed according to the criteria set out by American College of Rheumatology revised criteria in 1997 [33]. Disease activity was evaluated using the SLE Disease Activity Index (SLEDAI) and a cutoff of ≥8 was used to define active disease [34]. The SLE patients were aged 30 ± 6 years. For the arrays, six healthy subjects (3 males and 3 females) from local blood bank (150 mL) with a mean (±SD) age of 27 ± 8 years were recruited. Six patients with SLE (3 male and 3 female), who were hospitalized at the Clinical Unit of the Affiliated Drum Tower Hospital of Nanjing University Medical School, were recruited for whole blood (150 mL). For real-time quantitative PCR (qRT-PCR), independent samples were obtained; that is, ten healthy subjects (5 males and 5 females) from local blood bank (10 mL) with a mean (±SD) age of 26 ± 6 years and sixteen patients with SLE (8 male and 8 female), who were hospitalized at the Clinical Unit of the Affiliated Jinling Hospital of Nanjing University Medical School, were recruited for whole blood (10 mL). Although patients were on a variety of disease modifying agents, patients on high dose immunocytotoxic therapies or steroids were excluded from the study.

### 2.2. Sample Acquisition and Isolation of Human B Cells

Peripheral blood samples from study subjects were collected in heparinized tubes and immediately processed. Peripheral blood mononuclear cells (PBMCs) were isolated over Lymphocyte Separation Medium (LSM 1077) gradients for immediate use. Cells were kept at 4°C or on ice and cold buffers were employed to minimize alterations in gene expression during labeling and isolation. The purified B cells were prepared from PBMCs using negative selection by Pan B Cell Isolation Kit II from Miltenyi Biotec (Bergisch Gladbach, Germany). Immunofluorescence labeling for flow cytometry was performed by incubating isolated B cell with anti-CD19-PE antibodies (BD Biosciences, San Jose, CA) for 30 minutes and washing. Flow cytometry was performed using a FACSCalibur (BD Biosciences) with CellQuest software. Analysis was conducted with FloJo software. The purification of B cells over 95% was used in the study.

### 2.3. RNA Isolation and Microarray

The purified B cells were centrifuged in RNase-free tubes treated with Trizol reagent (Invitrogen, USA). Total RNA was extracted by using the RNeasy kit according to the instructions of the manufacture (Qiagen, Valencia, CA) and then RNA concentration was assessed by Nanodrop 2000 spectrophotometry (Thermo). RNA quality was determined by formaldehyde denaturation electrophoresis and only those samples with a 260 nm/280 nm optical density ratio (OD260/280) >1.8 and a total RNA concentration >1 mg/mL were submitted for hybridization to generate labeled targets.

Then the total RNA was used to synthesize double-strand cDNA (ds-cDNA) using an Invitrogen SuperScript ds-cDNA synthesis kit in the presence of oligo dT primers. The ds-cDNA was cleaned and labeled using NimbleGen One-Color DNA Labeling Kit in accordance with the manufacturer's protocol (NimbleGen Systems, Inc., Madison, WI). The labeled ds-cDNA was hybridized with NimbleGen 12 × 135 K Human Gene Expression Microarray consisting of 45,033 probes for human genes (Hybridization System-NimbleGen Sys-199 tems, Inc., Madison, WI). Following hybridization, washing was performed using the NimbleGen Wash Buffer kit (NimbleGen Systems, 201 Inc., Madison, WI). After being washed in an ozone-free environment, the processed slides were scanned with the Roche-NimbleGen MS200 confocal laser scanner (CapitalBio Corporation) using the recommended settings.

### 2.4. Analysis of Microarray Data

The obtained images were analyzed using NimbleScan software (version 2.5) for grid alignment and expression data analysis. Expression data were normalized through quantile normalization and the Robust Multichip Average (RMA) algorithm. All gene level files were imported into Agilent GeneSpring GX software (version 11.5.1) for further analysis. Genes showing minimal variation between defined phenotypes were excluded from analysis using a fold change filter set at 2-fold. Hierarchical clustering and principal components analysis using an uncentred correlation distance metric and average linkage clustering were performed in Cluster with visualization in Treeview. To evaluate the potential biological significance of the changes observed in the arrays, network analysis of the differentially expressed genes (DEGs) was performed, using the CapitalBio-Molecule Annotation System (MAS) software (http://bioinfo.capitalbio.com/mas3/). The *P* values used in the pathway and Gene Ontology (GO) analysis were calculated according to a hypergeometric distribution probability formula. The *P* or *q* values reflect the importance of the pathway or GO in the experimental results.

To determine the most significant biological functions and pathways of the DEGs, the two popular annotation databases: GO and Kyoto Encyclopedia of Genes and Genomes (KEGG), were applied. Both the up- and downregulated GO terms in female subjects were further divided into three subcategories: biological progress, cell component, and molecular function. Networks, which show relationships and interactions between DEGs and others that functionally interact with the genes, were generated and ranked in terms of their relevance, such as the number of participating genes, degree of connectivity, and size relative to the total number of network eligible genes.

### 2.5. Real-Time Quantitative PCR (qRT-PCR)

Total RNA was extracted from the isolated B cells. qRT-PCR was used to validate the selected data from the microarray experiments and to follow the expression of a subset of genes with the stimulation of estrogen. Total RNA was reverse-transcribed using the PrimeScript TM Reagent Kit (Takara) according to the manufacturer's instructions, with GAPDH serving as an endogenous control. The genes and their primer sequences used for the qRT-PCR assays are listed in Table 1. The cDNA samples were used for quantitative qRT-PCR analysis, which was performed using a 7300 qRT-PCR System (ABI) with a standard SYBR Green PCR protocol according to the manufacturer's instructions. The thermal cycling conditions included a first stage of 8 minutes at 95°C, a second stage corresponding to the 40 cycles of the PCR (for each cycle 95°C for 15 seconds and 65°C for 1 minute), and a third stage (rapid heating at 95°C, cooling at 60°C, and heating at 95°C). Each sample was run in triplicate alongside the endogenous control to normalize reactions. After completion of the PCR amplification, the relative fold change was calculated based on the 2^−ΔΔ^Ct method. In addition, total RNA of spleen B cells from mice was also extracted. Primer sequences were listed in Table 1.

### 2.6. Mice Ovariectomy and 17*β*-Estradiol Exposure

To determine whether the DEGs were related to estrogen, female mice were castrated. Briefly, BALB/cJ mice were housed under a 12 h light, 12 h dark cycle, with food and water ad libitum during the course of the experiment. Twelve BALB/cJ female mice were randomly divided into two groups: Ovx + vehicle (*n* = 6) and Ovx + E2 (*n* = 6). That is, mice were surgically ovariectomized (*n* = 12) after anesthesia using sodium pentobarbital. All animals were well owed to recover for two weeks and then received daily s.c. injections of either 17*β*-estradiol (E2) (100 mg/kg/day) which were dissolved in sesame oil or PBS every morning for 2 weeks. Mice were euthanized with a mixture of ketamine and medetomidine 24 h after receiving their last injection. Plasma was collected to determine hormone concentration using a commercial Estradiol EIA Kit according to the manufacturer's instruction.

### 2.7. Isolation of Mouse B Cells

For isolation of mouse B cells, the mouse spleen was dissociated with collagenase D treatment, and single-cell suspensions prepared from total spleen were incubated for 20 min at 4°C with Biotin-Antibody Cocktail (cocktail of monoclonal antibodies against CD43, CD4, and Ter-119) and Anti-Biotin MicroBeads. Then the cells were passed through the magnetized column twice, and the unlabeled cells were collected (MACS B cell isolation kit, Miltenyi, Germany). The purity of B cells measured by PE conjugated CD19 antibody was >95%. Purified B cells were used for the identification of some gene expression levels.

### 2.8. Statistic Analysis

All quantitative data were recorded as mean ± S.D. Comparisons between two groups were performed by Student's *t*-test. SPSS 13.0 (SPSS, Chicago, IL) was used for analyses, and statistical significance was defined as *P* < 0.05. Gene Ontology (GO) analysis of the differentially expressed genes was performed with GO categories (http://www.geneontology.org/).

## 3. Results 

### 3.1. B Cells Gene Expression Profiles Show DEGs between Healthy Female and Male

**Figure 1:**
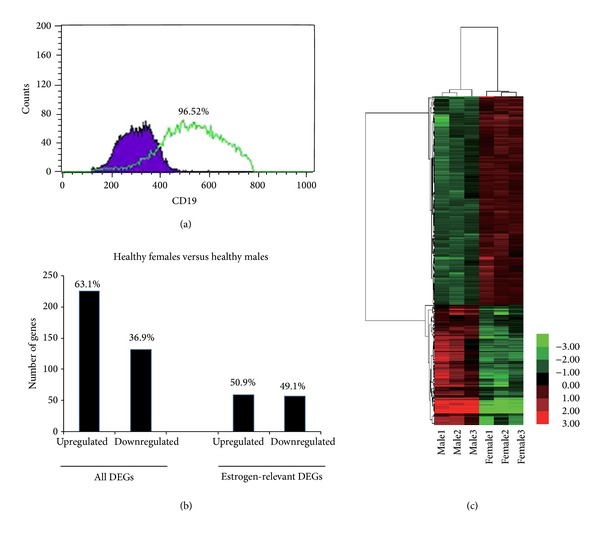
Gene profiling reveals differential expression genes between healthy females and males. (a) The purity of B cells was checked using CD19 antibody after selection using B cell isolation kit. (b) Hierarchical clustering of B cells of six healthy females and males using a list of 358 differentially expressed genes available on NimbleGen chips (adj. *P* value <0.05). The heat maps show the most highly changed gene expression (TOP100) in response to each stimulus. Each row in the heat maps represents a gene and each column represents a microarray sample. Red and green indicate upregulated and downregulated expression, respectively. All samples cluster in accordance with the gender of the participant, which shows a good homogeneous expression profile between healthy males' and females' groups. (c) The number which of genes displayed differential expression between healthy females and males. Estrogen-relevant DEGs were listed independently.

To elucidate whether the gene expression profiles of B cells were globally different between healthy female and male, B cells cDNA microarray analysis was conducted. Before cDNA microarray analysis, we first isolated and indentified the B cells from six healthy females and males. As shown in Figure 1(a), after selection with B Cell Isolation Kit, the purity of B cells reached to >95%. We defined the DEGs as a gene with a fold change ≥2 and a *P* value ≤0.05. According to the criteria, a total of 358 genes displayed differential expression between healthy female and male. Compared with male, 226 (63.1%, 226/358) and 132 (36.9%, 132/358) genes were found to be up- and downregulated in the female, respectively, Figure 1(c). As shown in Figure 1(b), hierarchical clustering analysis of the sample microarray data showed a good homogeneous expression profile between the two groups. The top twenty upregulated genes and twenty downregulated genes were displayed in Table 2. Moreover, CAV1, CD200R1, HRASLS2, MIA, TNFRSF17, CXCR3, and AQP3 among the top twenty upregulated genes as well as EIF1AY, DDX3Y UTY, IL7R, and CD3D among the top twenty downregulated genes are involved in immune function of organism. The reported function on these genes in detail will be discussed. Other genes function in immune responses was not clearly identified so far.

### 3.2. DEGs from Microarray Results of B Cells between Healthy Female and Male Confirmed by qRT-PCR

**Figure 2:**
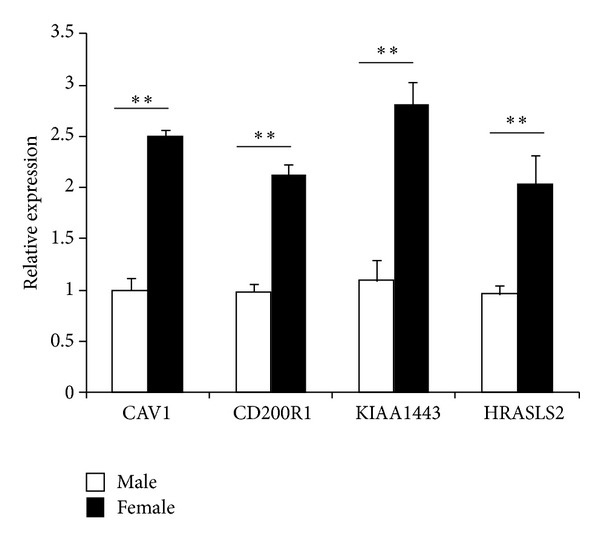
Validation of top 4 DEGs by qRT-PCR. The expression of 4 genes by the microarray was analyzed by QRT-PCR. Bar graph show real-time PCR expression of CAV1, CD200R1, KIAA1443, and HRASLS2 in B cells from healthy males and females. The data are presented as relative expression following normalization. Data represented mean ± SE. **P < 0.01 versus male.

Microarray analysis yields a large amount of data; therefore it is important to validate differential expression by independent methods. In order to verify the data obtained by microarray analysis, qRT-PCR was performed on 4 upregulated DEGs, that is, Cav1, CD200R1, KIAA1443, and HRASLS2. The expression ratios of the 4 genes, as determined by both microarray and qRT-PCR, were shown in Figure 2. Compared with males, the mean change folds of Cav1, CD200R1, KIAA1443, and HRASLS2 in female were 5.81, 5.52, 5.52, and 5.38 by microarray analysis, respectively (Table 2). qRT-PCR experiments in independent samples, that is, ten healthy subjects (5 males and 5 females), showed that mean fold changes (female/male) of Cav1, CD200R1, KIAA1443 and HRASLS2 were 2.53, 2.12, 2.90, and 2.24, respectively. The results of qRT-PCR for the 4 selected genes showed that the patterns of expression changes were consistent with those found by microarray analysis.

### 3.3. Gene Expression Profiles Show Estrogen-Relevant DEGs

It is well known that sex hormone, especially estrogen, has vital roles in immune-relevant biological functions. To refer to the estrogen-relevant DEGs from aforementioned 358 genes, correlation analysis and bibliographic retrieval between estrogen and DEGs were performed in KEGG and Pubmed databases. Interestingly, a total of 116 genes displayed possible correlation with estrogen. Compared with male, 59 (50.9%, 59/116) and 57 (49.1%, 57/116) genes were found to be up- and downregulated in the female, respectively (Figure 1(c)). The top twenty upregulated genes and twenty downregulated genes were displayed in Table 3. Moreover, CAV1, CD200R1, HRASLS2, MIA, TNFRSF17, CXCR3, AQP3, CCR9, IFNAR1, and CEACAM1 among the top twenty upregulated genes as well as EIF1AY, DDX3Y UTY, IL7R, IGFBP7, and GNLY among the top twenty downregulated genes are involved in immune function of organism, while other genes function in immune responses was not clearly identified.

### 3.4. GO Analysis for DEGs in Healthy Female and Male

**Figure 3:**
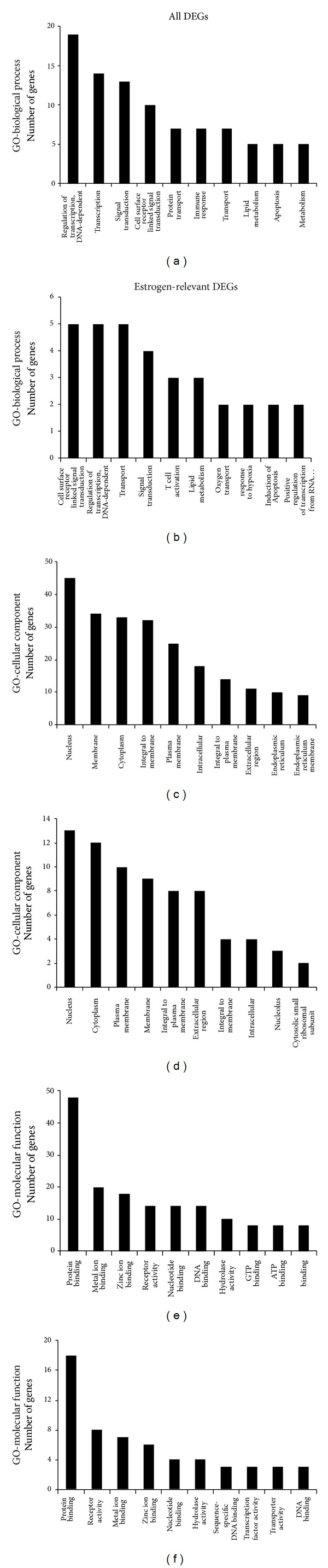
Lists of top ten GO terms for DEGs in healthy females and males. GO term covers three domains: biological process, cellular component, and molecular function. The left panels showed the GO terms for 358 DEGs in healthy females and males. The right panels showed the GO terms for 116 estrogen-relevant DEGs in healthy females and males. DEGs in healthy females' and males' comparison mainly perform DNA-dependent regulation of transcription (TR), biochemical reaction in nucleus, and protein binding and so forth. Estrogen-relevant DEGs widely participated in the cell surface receptor linked signal transduction, biochemical reaction in nucleus, protein binding and so forth.

To determine significant biological function classification and to reveal transcriptional correlations among genes of healthy subjects, the 358 significant genes were subjected to GO analysis. The GO analysis showed that several biological process categories were significantly overrepresented, including regulation of transcription (TR) (DNA-dependent), transcription, and signal transduction (Figure 3). In the cell component subcategory, the GO terms were mainly related to nucleus, membrane, and cytoplasm (Figure 3). Lastly, in the molecular function sub-category, protein binding, metal ion binding, zinc ion binding, and receptors activity were the significantly enriched items in GO analysis (Figure 3).

To reveal the main bioinformatics initiative to unify the representation of genes, the 116 estrogen-relevant DEGs were also subjected to GO analysis. The top ten enriched GO terms were shown in Figure 3, which showed that several biological process categories were significantly overrepresented, including cell surface receptor linked signal transduction, TR (DNA-dependent), and transport. In the cell component sub-category, the GO terms were mainly related to nucleus, cytoplasm, and plasma membrane, et al. (Figure 3). In the molecular function sub-category, protein binding, receptor activity, and metal ion binding were the significantly enriched items in GO analysis (Figure 3). Moreover, compared with the GO terms for all DEGs, these results clearly showed the partial difference of the GO terms for estrogen-relevant DEGs.

### 3.5. Signaling Pathway Enrichment Analysis

**Figure 4:**
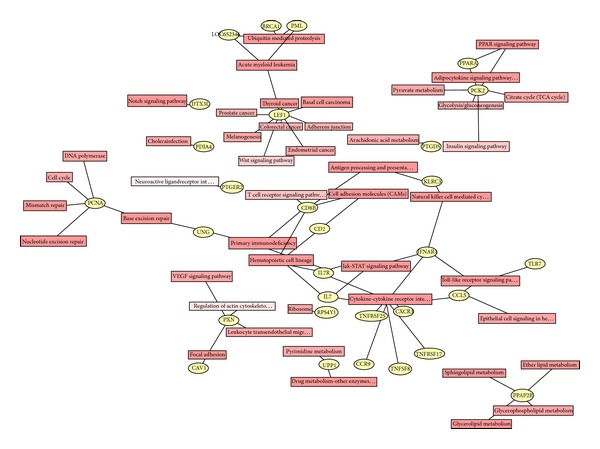
Enriched signaling pathways with significant association with estrogen-relevant DEGs. 116 estrogen-relevant DEGs were subjected to performing of the signaling pathway analysis based on KEGG databases. A group of 30 DEGs are found to be connected functionally, including LEF1, TNFRSF25, PDIA4, PXN, PTGDS, TNFRSF17, KLRC3, TNFSF8, PTGER2, CCR9, PCNA, UPP1, CD8B, CAV1, UNG, PPAP2B, CD2, LOC652346, IFNAR1, BRCA1, IL7R, PML, IL7, PPARA, CCL5, PCK2, CXCR3, DTX3L, RPS4Y1, and TLR7. Blue stars indicate that these 9 DEGs are highly related to the immunoreactions.

To identify the significantly enriched signaling pathways, KEGG, Biocarta, and Genmapp databases were separately employed. The top ten enriched signaling pathways for all DEGs in three databases were listed in Table 4. The KEGG pathways analysis revealed that pathways associated with immune functions were highly significantly, including cytokine-cytokine receptor interaction and natural killer cell mediated cytotoxicity (Table 4 and Figure  S1 (see Supplementary Material available online at http://dx.doi.org/10.1155/2014/814598)). Moreover, estrogen-relevant DEGs were also subjected to performing of standard signaling pathway enrichment analysis, and the top ten enriched signaling pathways in three databases were listed in Table 5. The KEGG pathways analysis revealed that pathways associated with estrogen were cytokine-cytokine receptor interaction, hematopoietic cell lineage, and primary immunodeficiency (Table 5, Figure 4). Interestingly, a group of 30 DEGs are found connected functionally, including LEF1, KLRC3, PCNA, IFNAR1, IL7R, CCL5, CXCR3, CAV1, and TLR7 (Figure 4).

We also identified the significantly enriched signaling pathways for all DEGs or estrogen-relevant DEGs based on Biocarta, and Genmapp databases. As expected, several immune-related pathways were also significantly overrepresented by Biocarta pathways analysis. Top ranked Biocarta pathways included NO_2_-dependent IL12 pathway in NK cells, Ras-independent pathway in NK cell mediated cytotoxicity, and IL12/Stat4-dependent signaling pathway in Th1 development (Table 4). Moreover, the Biocarta pathways analysis revealed that pathways associated with estrogen were NO_2_-dependent IL 12 pathway in NK cells, IL-7 Signal Transduction, and Dendritic cells in regulating TH1 and TH2 Development and so forth (Table 5). Furthermore, sixty-nine differentially expressed genes were identified within the Genmapp pathways, including humoral immune response, inflammatory response, and humoral defense mechanism pathway (Table 4), while the Genmapp pathways analysis revealed that pathways associated with estrogen were Tissue-specific-Hs-1-Tissue-Blood and Lymph, GO_Samples-Biological process-cell motility, and GO_Samples-Biological process-locomotion (Table 5). It is thus obvious that the observations obtained in this study had support of the hypothesis that a baseline gender-associated difference in B cell signature and function existed.

### 3.6. Genes Were Differentially Expressed in B Cells of SLE Male and Female

To observe whether the baseline gender difference of immune function contribute to autoimmune diseases, the DEGs in B cells of SLE males and females were determined by microarray. Surprisingly, only 26 gene expressions were downregulated in SLE females versus SLE males, of which most genes were linked to Y chromosome (Supplementary Table  1). Moreover, the list of DEGs and fold changes were submitted for bioinformatic function analyses. The KEGG pathways analysis revealed that pathways associated with B cells of SLE males were ribosome-relevant signaling pathways, indicating the abundant protein synthesis in abnormal B cells (Supplementary Table  2). The Genmapp pathways analysis 27 enriched signaling pathways, including biological process spermatogenesis, cellular component male gamete generation, and cellular component gametogenesis (Supplementary Table  2). In addition, the top ten enriched signaling pathways with significant association with DEGs or with estrogen-relevant DEGs (SLE females/SLE males) were provided in Supplementary Tables  3 and 4.

### 3.7. DEGs with Different Expression Ratios between Healthy Subjects and SLE Patients

Although our above described results showed a baseline gender-associated gene difference in B cells from both healthy subjects and SLE patients between females and males, it is very difficult to figure out the specific genes responsible for disruption of immune balance of B cells in SLE patients. Therefore, the B cell signatures of healthy subjects and SLE patients were synthetically compared. To determine the DEGs between healthy subjects and SLE patients, the expression ratios of genes (female/male) determined by microarray were analyzed to exclude those genes which were downregulated (female/male) in both healthy subjects and SLE patients. Strikingly, the results showed that, among the downregulated expressions of 26 genes in SLE females versus SLE male, only eight downregulated expression genes have nonsignificant difference between healthy females and healthy males (Table 6). Moreover, among the downregulated expressions of 8 genes, except for the 5 Y-chromosome-related genes or variants, only 3 DEGs between healthy subjects and SLE patients deserved to be further studied. Interestingly, the three genes (LTF, CAMP, and DEFA4, encoding lactoferrin, cathelicidin, and *α*-defensin 4) were closely related to immune function and autoimmune diseases [35–37].

### 3.8. Validation of LTF, CAMP, and DEFA4 in B Cells from SLE Patients by qRT-PCR

**Figure 5:**
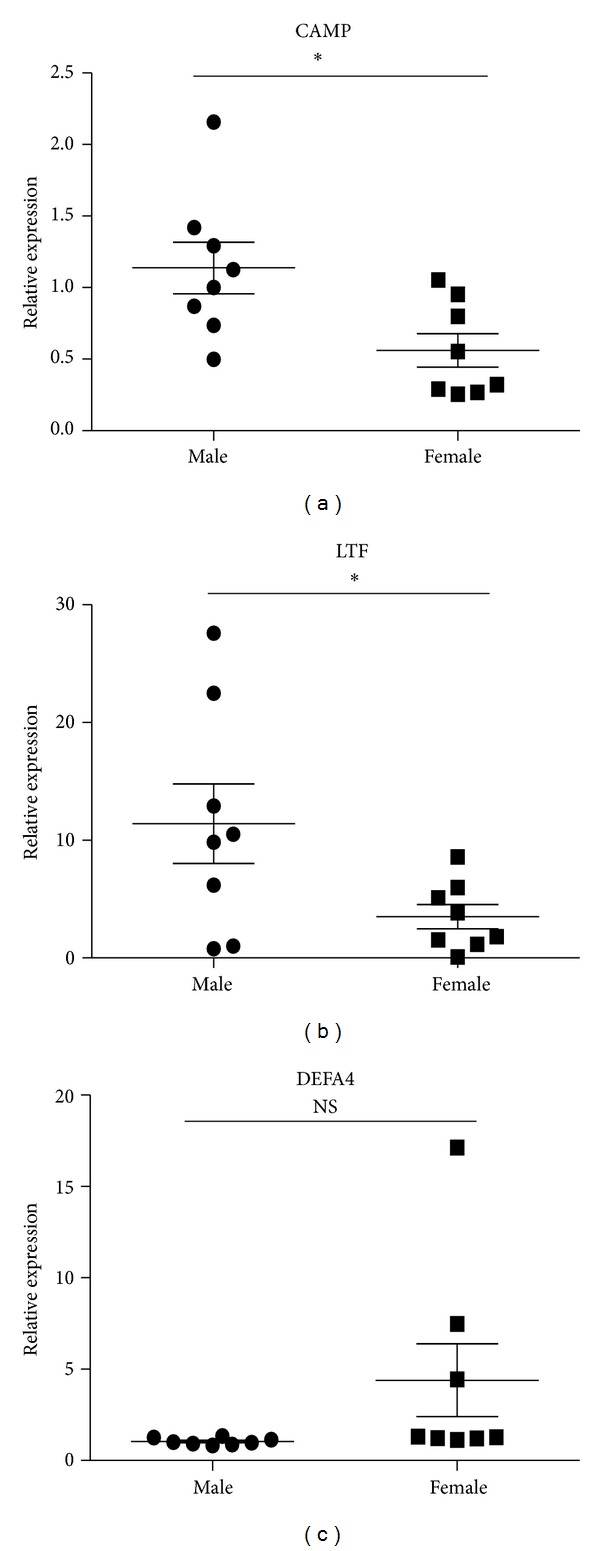
Expression levels of CAMP, LTF, and DEFA4 measured by qPCR in B cells from SLE patients. B cells were purified from eight male SLE patients and eight female SLE patients. Then the expression of CAMP, LTF, and DEFA4 was checked by qPCR. The data are presented as relative expression following normalization. Asterisks represent significant difference to the male (*P < 0.05). NS: *P* > 0.05.

Consistent with decreased expression ratios of microarray (female/male), results of qRT-PCR revealed that LTF and CAMP were decreased in female SLE patients. The expression ratio of DEFA4 between female and male SLE patients by microarray was 0.4241. However, QRT-PCR analysis showed that there was no significant difference in DEFA4 between female and male SLE patients in independent samples of sixteen patients with SLE (8 male and 8 female) (Figure 5). Compared with results of SLE patients, the expression ratios of these three genes in female healthy subjects also were of greater magnitude.

### 3.9. Mouse Plasma 17*β*-Estradiol Levels and Its Effect on the Expression of LTF, CAMP, and DEFA4

**Figure 6:**
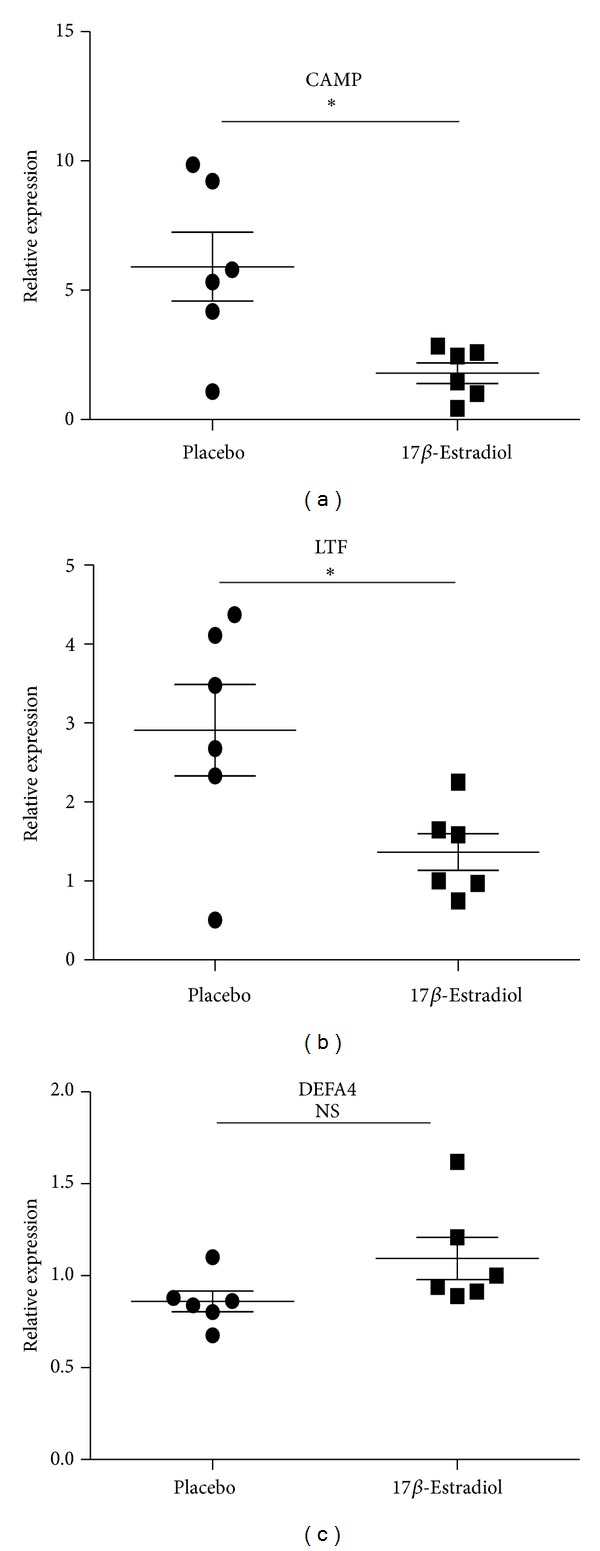
Expression levels of CAMP, LTF, and DEFA4 measured by qPCR in B cells from castrated and sham-castrated mice. B cells were purified from six male SLE patients and eight female SLE patients. Then the expression of CAMP, LTF, and DEFA4 was checked by qPCR. The data are presented as relative expression following normalization. Asterisks represent significant difference to the male (*P < 0.05). NS: *P* > 0.05.

Gender differences in the immune function predisposed women to immune-related diseases that were exacerbated following menopause. Sex hormone, especially estrogen, had important effects on the immune function. To determine whether estrogen has effect on the expression of LTF, CAMP, and DEFA4, the female mice were ovariectomied and then received daily s.c. injections of either 17*β*-estradiol (E2) (100 mg/kg/day) which were dissolved in sesame oil or PBS every morning for 2 weeks. The B cells from both ovariectomized mice with exposure to E2 and sham-ovariectomied mice were isolated and expressions of LTF, CAMP, and DEFA4 were detected by qRT-PCR. In agreement to results of SLE patients, the expressions of LTF and CAMP were significantly lower in female ovariectomied mice with exposure to E2 than sham-ovariectomied mic, while the DEFA4 levels showed no significant difference between castrated and sham-castrated mice. Compared to the results of healthy human subjects, the expression ratios of these three genes decreased significantly in mice (sham-ovariectomied mice/ovariectomied mice with exposure to E2) (Figure 6). In addition, the results detected by ELISA confirmed that plasma E2 concentration in Ovx + E2 group mice was 35.23 ± 3.64 pg/mL (*n* = 6). E2 could not be detected in Ovx + vehicle treated group. The normal physiological range of plasma E2 concentration in the mouse was reported to be between 5 and 50 pg/mL. These results suggest the effect of estrogen on expression of LTF, CAMP, and DEFA4.

## 4. Discussion 

Clinical and basic science investigations have shown important sex differences in health and the onset and the progression of the disease as well as in pharmacological research [1–5, 7–10]. The DEGs revealed sex-related differences in mRNA expression, specific pathways and networks of kidney [4, 6]. Some evidences also showed that cells of males and females may display several different features and behaviours [16, 17]. However, specific genes responsible for gender differences in immunity remain poorly understood. In our present study, gene expression profiles showed DEGs of B cells between healthy female and male. DEGs was defined as a statistically significant difference with a fold-change ≥2 and a *P* value ≤0.05. A total of 358 DEGs was displayed between healthy female and male. Compared with male, 226 and 132 genes were found to be up- and downregulated in the female, respectively. Four top upregulated DEGs including Cav1, CD200R1, KIAA1443 and HRASLS2 were confirmed by qRT-PCR. Moreover, CAV1, CD200R1, HRASLS2, MIA, TNFRSF17, CXCR3, and AQP3 among the top twenty upregulated genes as well as EIF1AY, DDX3Y UTY, IL7R and CD3D among the top twenty downregulated genes are involved in immune function of organism, while other genes function in immune responses was not clearly identified.

Hewagama group used microarray to compared T-cell gene expression between healthy men and women. The results showed that 1953 differentially expressed genes were identified [38]. Sankaran-Walters also found that healthy women had higher levels of immune activation and inflammation-associated gene expression in gut mucosal lymphoid tissue [10]. As is well known, B cells belong to a vital part of the immune system and their principal functions are to make antibodies, perform antigen processing and produce cytokine. It was reported that CAV1 present in immune cells may be involved in regulation of the cell signaling and inflammatory response [39] as well as the Jak-Stat signaling pathway [40] and tumour-induced immunosuppression [41]. The CD200/CD200R axis also has been reported to be important in regulating the immune responses. CD200 can regulate the activation threshold of inflammatory immune responses, polarize cytokine production, and maintain immune homeostasis [42]. HRASLS2 exhibit tumor-suppressing activities and may be involved in the phospholipid metabolism with different physiological roles [43]. MIA contributes to immunosuppression frequently seen in malignant melanomas by binding to integrin *α*4*β*1 expressed by leukocytes and thus inhibiting cellular antitumor immune response [44]. AQP3 mediated H_2_O_2_ uptake is required for chemokine-dependent T cell migration in sufficient immune response [45]. The minor histocompatibility antigen UTY may be a promising target to further improve graft-versus-leukemia immune reactions after allogeneic stem cell transplantations [46]. CD3D gene encodes CD3*δ* protein, which is involved in TCR signaling [47]. But the function of many genes in B cell was not clearly identified.

Of note, ER*α* is known to colocalize with CAV1 protein and CAV1 protein stimulates the translocation of ER*α* to the plasma membrane, which facilitates the activation of the PI3 kinase pathway [48]. Recent study found that CD200 and CD200R1 expression and function were abnormal in SLE and may contribute to the immunologic abnormalities in SLE [49]. Interestingly, TNFRSF17 is preferentially expressed in mature B cells and may be important for B cell development and autoimmune response. TNFRSF17 has been shown to interact with the B cell activating factor TNFSF13B and lead to NF-*κ*B and MAPK8/JNK activation [50]. Although CXCR3 is expressed primarily on activated T lymphocytes and NK cells, the inflammation-related CXCR3 chemokine receptor is uniquely expressed by CD138(high) MHCII(+) plasma cells, which encompass both short- and long-lived cells and mostly produce IgG autoantibodies in NZB/W mice [51]. The genes (EIF1AY and DDX3Y) are located on the nonrecombining region of the human Y chromosome and were identified to be associate with immunology, RNA metabolism, vesicle fusion, and angiogenesis [52]. We thus presume that the upregulated DEGs such as Cav1, CD200R1, TNFRSF17 and CXCR3 and downregulated DEGs such as EIF1AY and DDX3Y in healthy female may be involved in gender predominance of some immune diseases.

Humoral immune responses are sexually dimorphic. Female individuals are more likely than male individuals to produce autoreactive antibodies of pathogenic potential. Moreover, the gonadal steroids modulate humoral immune responses including B cell development, function and immune tolerance [25]. We here found that estrogen-relevant 59 up- and 57 downregulated DEGs in the female compared with male. Moreover, the upregulated DEGs (CAV1, CD200R1, HRASLS2, MIA, TNFRSF17, CXCR3, AQP3, CCR9, IFNAR1, and CEACAM1) and downregulated DEGs (EIF1AY, DDX3Y UTY, IL7R, IGFBP7, and GNLY) may be involved in immune function of organism. Except for above discussed DEGs, the chemokine receptor CCR9 is one of the key molecules in leukocyte homing to gut mucosa [53]. IGFBP7 is reported to induce pregnancy failure by shifting uterine cytokines to Th1 type dominance [54]. GNLY and GZMH were demonstrated to be related to apoptosis [55, 56]. Of note, all interferons are considered to signal via the heterodimeric IFNAR1-IFNAR2 complex, but IFN-*β* uniquely and specifically ligate to IFNAR1. The IFNAR1-IFN-*β* complex transduced signals that modulated expression of a distinct set of genes, suggesting that IFNAR1-IFN-*β* signaling is pathologically relevant [57]. Type I IFNs (IFN-I) have also been implicated in the pathogenesis of lupus both in patients and in several murine models of disease. IFNAR1 regulated anticytoplasmic and anti-RNA specificities and its deficiency prevented the exacerbation of clinical disease in this lupus model [58]. In addition, CEACAM1 may act as an inhibitory B cell coreceptor and promote activation of B cells by anti-sIgM or anti-CD19 antibodies in a PI3 K-dependent manner [59]. It is thus evident that DEGs especially Cav1, CD200R1, TNFRSF17, CXCR3, IFNAR1, and CEACAM1 and downregulated DEGs such as IL7R, EIF1AY, and DDX3Y of B cells between healthy female and male may be related to estrogen level in these subjects.

Strikingly, signaling pathway analysis based on KEGG databases showed that the highly integrated network for estrogen-relevant DEGs covers 30 genes, including A group of 30 DEGs are found connected functionally, including LEF1, KLRC3, PCNA, IFNAR1, IL7R, CCL5, CXCR3, CAV1, TLR7. These above mentioned DEGs are widely reported in immune response and its association with diseases [60–64]; that is, LEF1 contributed to the survival and proliferation of pro-B cells in response to extracellular signals. Numerous genes may enhance immune function and responsiveness including activating receptors such as KLRC3. PCNA was originally identified as an antigen that is expressed in the nuclei of cells during the DNA synthesis phase of the cell cycle. B cells are activated through IFNAR. IL-7R is required for development and maintenance of the immune system. B cell production of CCL5 has important autocrine effects. B cells acquire CXCR3 that causes their migration to inflammatory foci. Moreover, estrogens readily diffuse across the cell membrane and subsequently bind to and activate estrogen receptors which in turn modulate the expression of many genes. The abnormal expression of estrogen or its receptors frequently leads to immunological diseases, including SLE. These findings, along with our above results, suggest that a baseline gender-associated difference of immune-related genes may exist in both healthy and SLE persons.

SLE is the prototypic systemic autoimmune disease and possesses a gender-dependent incidence characterized by a male/female ratio of 1 : 9 [65, 66]. Although our above described results indicated a gender-associated gene difference of B cells from both healthy subjects and SLE patients between female and male, it is very difficult to figure out the specific genes responsible for disruption of immune balance of B cells in SLE patients. Therefore, the B cell signatures of healthy subjects and SLE patients were synthetically compared. We found that only 3 DEGs between healthy subjects and SLE patients need to be analyzed. The three genes are LTF, CAMP and DEFA4, which coded lactoferrin, cathelicidin and *α*-defensin 4, respectively. In the present study, the expression of lactoferrin and cathelicidin in the B cells from female was more decreased than that from male. Lactoferrin and cathelicidin belong to host defence peptides (HDPs), which are critical effectors of both innate and adaptive immunity. Mechanistic studies revealed that the role of HDPs in immunity is very complex and involves various receptors, signalling pathways and transcription factors [67, 68]. Thus, it is evident that gender difference indeed exists in DEGs of B cells including LTF and CAMP genes.

Since sex hormones, especially estrogen, had immune-modulatory properties and contributed to sex-bias susceptibility to some immune diseases [69], the effects of estrogen on LTF, CAMP and DEFA4 were also explored in the present study. The levels of estrogen decreased more significantly in the castrated female mice than those of sham-castrated mice. Interestingly, the expressions of LTF and CAMP also increased in castrated female mice. The replication results were consistent with those of male and female SLE patients. However, the regulation of the immune response is a complex interplay of multiple factors, but it is becoming increasingly clear that sex steroid hormones, and in particular the principle female sex steroid estrogen, exert potent effects on the immune response.

In addition, it is surprising that only 26 gene expressions were downregulated in SLE female versus SLE male, of which most genes were linked to Y chromosome, when we defined the differentially expressed gene as a gene with a fold-change ≥2 and a *P* value ≤0.05. This suggests that a gender difference of immunity may be related to some gene activation, which may be attributed to threshold of B cell signaling activation to reach some degree to induce the onset and the progression of SLE in male which is similar to female SLE. As is analyzed above, some gene expression and activation of signaling pathway were higher in SLE female versus SLE male. Thus immune equilibrium may be necessary to prevent the occurrence and development of SLE in female and male. This also may be one of the factors which result in a male/female ratio of 1 : 9.

The study may provide new understanding of the influence of gonadal steroid hormones on the humoral immune system, especially B cell function and its molecular basis. The gender differences in global gene expression of B cells were existent between healthy female and male subjects. This difference may be related to estrogen. These findings suggest that the diagnosis and treatment of these immune-related diseases should consider the baseline gender-related differences. Our study highlights the need for more detailed analysis of the effects of gender differences in immune responses.

## Supplementary Material

The differential gene expressions (DEGs) in B cells of SLE males and females were determined by microarray. Only 26 gene expressions were down-regulated in SLE females versus SLE males, which of most genes were linked to Y chromosome (Supplementary Table 1). And eight DEGs (CYorf15A, DEFA4, NLGN4Y, CAMP, KIAA1666 and three LTF varients) have non-significant different between healthy females and healthy males (Supplementary Table 1).
The KEGG pathways analysis revealed that pathways associated with B cells of SLE males were Ribosome-relevant signaling pathways, indicating the abundant protein synthsis in abnormal B cells (Supplementary Table 2). The Genmapp pathways analysis 27 enriched signaling pathways, including biological process spermatogenesis, cellular component male gamete generation, and cellular component gametogenesis, et al (Supplementary Table 2). Top ten enriched signaling pathways with significant association with DEGs or with estrogen-relevant DEGs (SLE females/SLE males) were provided in Supplementary Table 3 and Supplementary Table 4.
358 DEGs in B cells of healthy females' and males' comparison were subjected to perform the signaling pathway analysis based on KEGG databases. A group of 65 DEGs are found connected functionally, including the estrogen-relevant DEGs described in Figure 4 (Figure S1).

## Figures and Tables

**Table 1:** Sequence of primers used for real-time quantitative PCR with SYBR Green.

Gene	Forward	Reverse
Human		
CAV1	GCAGAACCAGAAGGGACA	GCCAAAGAGGGCAGACA
CD200R1	TCGTGGGATTCATTTGG	ATGCCTTCACCTTGTTTGT
KIAA1443	CGGCGTGAGGATTACCA	AAGGAGCGGGTTGATGG
HRASLS2	CAACTACAGGGTCAATAACAAGC	GGTCAGCGAATAAGGCAACT
LTF	TGCCCAACAGCAACGAG	CCTTATCCATCCGAGACACC
CAMP	GCTAACCTCTACCGCCTCC	CCAGCCCGTCCTTCTTG
DEFA4	TCCAGGCAAGAGGTGATG	ATGAGGCAGTTCCCAACA
Mouse		
LTF	CATTGGCTTTGTGAGGGTT	CAGTCTTCCGTGGTGGG
CAMP	CTTCAACCAGCAGTCCCTAG	TTGCCACATACAGTCTCCTTC
DEFA4	CAGGCTGATCCTATCCAA	TGGACAAGTCCCACGAA

**Table 2:** The top 20 upregulated and downregulated differentially expressed genes between healthy females and males (fold change = ratio of healthy females/healthy males).

Gene ID	Gene	Gene description	*q* value (%)	Fold Change
Upregulated				
AF172085	CAV1	Caveolin 1, caveolae protein	2.67317	5.8121
BC069661	CD200R1	CD200 receptor 1	4.39788	5.5199
NM_020834	KIAA1443	KIAA1443	0	5.5175
NM_017878	HRASLS2	HRAS-like suppressor 2	4.21816	5.3753
NM_138806	CD200R1	CD200 receptor 1	4.95631	5.1925
BC082246	CAV1	Caveolin 1, caveolae protein	2.8169	4.8298
NM_001001712	LCN10	Lipocalin 10	3.77926	4.3216
NM_006533	MIA	Melanoma inhibitory activity	4.21816	4.2646
NM_016095	Pfs2	DNA replication complex GINS protein PSF2	4.95631	4.2172
BC058291	TNFRSF17	Tumor necrosis factor receptor superfamily, member 17	4.95631	3.9719
BC028973	MKKS	McKusick-Kaufman syndrome	4.39787	3.9554
AF469635	CXCR3	Chemokine (C-X-C motif) receptor 3	0	3.9458
XM_927420	LOC653154	Similar to brain protein 16	4.21816	3.8507
NM_004650	PNPLA4	Patatin-like phospholipase domain containing 4	0	3.8467
NM_004925	AQP3	Aquaporin 3 (Gill blood group)	3.16872	3.7642
BC014550	NT5DC2	5′-nucleotidase domain containing 2	0	3.722
NM_022908	NT5DC2	5′-nucleotidase domain containing 2	2.48701	3.6865
BC013870	GOLPH3L	Golgi phosphoprotein 3-like	3.77926	3.6842
NM_178518	GOLPH3L	Transmembrane protein 102	0	3.6597
NM_001504	CXCR3	Chemokine (C-X-C motif) receptor 3	2.14645	3.628
Downregulated				
NM_004653	SMCY	Smcy homolog, Y-linked	0	0.0016
D87072	SMCY	Smcy homolog, Y-linked	0	0.002
NM_032576	CYorf15B	Chromosome Y open reading frame 15B	0	0.0022
NM_004654	USP9Y	Ubiquitin specific peptidase 9, Y-linked (fat facets-like, drosophila)	0	0.0032
NM_004681	EIF1AY	Eukaryotic translation initiation factor 1A, Y-linked	0	0.0042
NM_004660	DDX3Y	DEAD (Asp-Glu-Ala-Asp) box polypeptide 3, Y-linked	0	0.0046
NM_001008	RPS4Y1	Ribosomal protein S4, Y-linked 1	0	0.0056
BC100905	RPS4Y1	Ribosomal protein S4, Y-linked 1	0	0.0059
NM_003411	ZFY	Zinc finger protein, Y-linked	0	0.0076
NM_182659	UTY	Ubiquitously transcribed tetratricopeptide repeat gene, Y-linked	0	0.0078
BC035312	CYorf15B	Chromosome Y open reading frame 15B	0	0.0083
NM_001005852	CYorf15A	Chromosome Y open reading frame 15A	0	0.0148
NM_007125	UTY	“Ubiquitously transcribed tetratricopeptide repeat gene, Y-linked”	0	0.0163
NM_182660	UTY	“Ubiquitously transcribed tetratricopeptide repeat gene, Y-linked”	0	0.0176
AF332239	TTTY10	“Testis-specific transcript, Y-linked 10”	0	0.0265
BC034942	DDX3Y	“DEAD (Asp-Glu-Ala-Asp) box polypeptide 3, Y-linked”	0	0.0388
BC067537	IL7R	Interleukin 7 receptor	0.6716099	0.0418
NM_002185	IL7R	Interleukin 7 receptor	0	0.0523
BC070321	CD3D	“CD3d molecule, delta (CD3-TCR complex)”	0	0.0614
NM_000954	PTGDS	Prostaglandin D2 synthase 21 kDa (brain)	0	0.0632

**Table 3:** The top 20 upregulated and downregulated estrogen-relevant DEGs between healthy females and males (fold change = ratio of healthy females/healthy males).

Gene ID	Gene	Gene description	*q* value (%)	Fold Change
Upregulated				
AF172085	CAV1	Caveolin 1, caveolae protein	2.67317	5.8121
BC069661	CD200R1	CD200 receptor 1	4.39788	5.5199
NM_020834	KIAA1443	KIAA1443	0	5.5175
NM_017878	HRASLS2	HRAS-like suppressor 2	4.21816	5.3753
NM_138806	CD200R1	CD200 receptor 1	4.95631	5.1925
BC082246	CAV1	Caveolin 1, caveolae protein	2.8169	4.8298
NM_001001712	LCN10	Lipocalin 10	3.77926	4.3216
NM_006533	MIA	Melanoma inhibitory activity	4.21816	4.2646
BC058291	TNFRSF17	Tumor necrosis factor receptor superfamily, member 17	4.95631	3.9719
BC028973	MKKS	McKusick-Kaufman syndrome	4.39788	3.9554
AF469635	CXCR3	Chemokine (C-X-C motif) receptor 3	0	3.9458
NM_004925	AQP3	Aquaporin 3 (Gill blood group)	3.16872	3.7642
NM_001504	CXCR3	Chemokine (C-X-C motif) receptor 3	2.14645	3.628
NM_006641	CCR9	Chemokine (C-C motif) receptor 9	4.39788	3.5421
AY030238	LQK1	LQK1 hypothetical protein short isoform	2.48701	3.369
XM_931870	LQK1	LQK1 hypothetical protein short isoform	2.14645	3.2506
BC009196	PPAP2B	Phosphatidic acid phosphatase type 2B	4.95631	3.1285
NM_000629	IFNAR1	“Interferon (alpha, beta and omega) receptor 1”	1.77977	3.1025
D12502	CEACAM1	Carcinoembryonic antigen-related cell adhesion molecule 1 (biliary glycoprotein)	4.39788	2.9871
NM_198990	NAPE-PLD	N-acyl-phosphatidylethanolamine-hydrolyzing phospholipase D	2.14645	2.966
Downregulated				
NM_004681	EIF1AY	Eukaryotic translation initiation factor 1A, Y-linked	0	0.0042
NM_004660	DDX3Y	DEAD (Asp-Glu-Ala-Asp) box polypeptide 3, Y-linked	0	0.0046
NM_001008	RPS4Y1	Ribosomal protein S4, Y-linked 1	0	0.0056
BC100905	RPS4Y1	Ribosomal protein S4, Y-linked 1	0	0.0059
NM_003411	ZFY	Zinc finger protein, Y-linked	0	0.0076
BC034942	DDX3Y	DEAD (Asp-Glu-Ala-Asp) box polypeptide 3, Y-linked	0	0.0388
BC067537	IL7R	Interleukin 7 receptor	0.67161	0.0418
NM_002185	IL7R	Interleukin 7 receptor	0	0.0523
NM_000954	PTGDS	Prostaglandin D2 synthase	0	0.0632
BC041463	PTGDS	Prostaglandin D2 synthase	0	0.0656
CR610092	PTGDS	Prostaglandin D2 synthase 21 kDa (brain)	0	0.0693
XM_371783	LOC202134	Hypothetical protein LOC202134	2.30889	0.0905
NM_002261	KLRC3	“Killer cell lectin-like receptor subfamily C, member 3”	1.37788	0.0925
NM_001553	IGFBP7	Insulin-like growth factor binding protein 7	0	0.0937
NM_004931	CD8B	CD8b molecule	0	0.0956
NM_173663	NY-REN-7	NY-REN-7 antigen	1.03928	0.0996
BC023576	GNLY	Granulysin	0.84751	0.1
NM_001767	CD2	CD2 molecule	0	0.1021
AY232658	GZMH	“Granzyme H (cathepsin G-like 2, protein h-CCPX)”	0	0.1039
L14542	KLRC3	“Killer cell lectin-like receptor subfamily C, member 3”	2.81690	0.1063

**Table 4:** Top ten enriched signaling pathways with significant association with DEGs (healthy females/males).

	Number of genes	*P* values
Kegg pathways		
Cytokine-cytokine receptor interaction	10	1.01*E* − 08
Natural killer cell mediated cytotoxicity	8	1.25*E* − 08
Hematopoietic cell lineage	7	1.07*E* − 08
Antigen processing and presentation	6	3.60*E* − 07
Primary immunodeficiency	5	7.75*E* − 08
Ubiquitin mediated proteolysis	5	7.43*E* − 05
Jak-STAT signaling pathway	5	1.24*E* − 04
Graft-versus-host disease	4	8.79*E* − 06
Acute myeloid leukemia	4	3.43*E* − 05
Toll-like receptor signaling pathway	4	2.89*E* − 04
Biocarta pathways		
NO2-dependent IL 12 Pathway in NK cells	5	2.00*E* − 10
Ras-independent pathway in NK cell mediated cytotoxicity	5	7.54*E* − 10
IL12 and Stat4 dependent signaling pathway in Th1 development	4	3.22*E* − 07
Stathmin and breast cancer resistance to antimicrotubule agents	3	4.63*E* − 06
T cytotoxic cell surface molecules	3	4.63*E* − 06
T helper cell surface molecules	3	4.63*E* − 06
Lck and Fyn tyrosine kinases in initiation of TCR activation	3	6.15*E* − 06
CTL mediated immune response against target cells	3	6.15*E* − 06
IL 17 signaling pathway	3	1.55*E* − 05
T cell receptor and CD3 complex	2	1.40*E* − 04
Genmapp pathways		
Hs_1-Tissue-Blood_and_Lymph	9	1.95*E* − 09
Cell surface receptor linked signal transduction	8	4.73*E* − 09
Cellular_process-Hs_T-Cell-Receptor_NetPath_11	8	1.05*E* − 08
Humoral immune response	7	5.73*E* − 07
Inflammatory response	7	1.94*E* − 06
Transmembrane receptor activity	6	7.18*E* − 07
Humoral defense mechanism (sensu Vertebrata)	6	1.40*E* − 06
Isomerase activity	6	1.99*E* − 06
Lipid metabolism	6	6.51*E* − 06
Hs_TNF-alpha-NF-kB_NetPath_9	6	2.65*E* − 05

**Table 5:** Top ten enriched signaling pathways with significant association with estrogen-relevant DEGs (healthy females/males).

	Number of genes	*P* Value
KEGG pathways		
Cytokine-cytokine receptor interaction	9	2.89*E* − 11
Hematopoietic cell lineage	4	3.84*E* − 06
Primary immunodeficiency	3	1.04*E* − 05
Acute myeloid leukemia	3	5.08*E* − 05
Toll-like receptor signaling pathway	3	2.59*E* − 04
Ubiquitin mediated proteolysis	3	6.39*E* − 04
Jak-STAT signaling pathway	3	8.76*E* − 04
Base excision repair	2	8.69*E* − 04
Adipocytokine signaling pathway	2	0.002977
PPAR signaling pathway	2	0.003244
Biocarta pathways	
NO2-dependent IL 12 Pathway in NK cells	2	9.27*E* − 05
IL-7 signal transduction	2	1.09*E* − 04
Dendritic cells in regulating TH1 and TH2 development	2	2.14*E* − 04
Mitochondrial carnitine palmitoyltransferase (CPT) system	1	0.004794
Basic mechanism of action of PPARa, PPARb(d), and PPARg and effects on gene expression	1	0.004794
The role of eosinophils in the chemokine network of allergy	1	0.005989
BRCA1-dependent Ub-ligase activity	1	0.008375
Arrestins in GPCR desensitization	1	0.009566
IFN alpha signaling pathway	1	0.010756
Regulation of PGC-1a	1	0.010756
Genmapp pathways	
Tissue-specific-Hs_1-Tissue-Blood_and_Lymph	6	4.16*E* − 08
GO_Samples-Biological process-cell motility	5	2.04*E* − 06
GO_Samples-Biological process-locomotion	5	2.04*E* − 06
GO_Samples-Biological process-locomotory behavior	5	2.36*E* − 06
Contributed-metabolic process-Hs Prostaglandin synthesis regulation	3	7.19*E* − 06
GO_Samples-Molecular function-isomerase activity	4	1.33*E* − 05
GO_Samples-Biological process-cell surface receptor linked signal transduction	4	1.47*E* − 05
GO_Samples-Biological process-humoral immune response	4	3.76*E* − 05
GO_Samples-Biological process-inflammatory response	4	7.64*E* − 05
GO_Samples-Biological process-response to chemical substance	4	1.07*E* − 04

**Table 6:** Eight down-regulated expression genes in SLE females versus SLE males, which have nonsignificant difference between healthy females and healthy males.

Gene ID	*q* value (%)	Fold change	Chromosomal	Symbol	Full name
NM_001005852	0	0.0102	chrY	CYorf15A	Chromosome Y open reading frame 15A
BC093959	4.9773348	0.0635	chr8	DEFA4	Defensin, alpha 4, corticostatin
BC015823	0	0.0849	chr3	LTF	Lactotransferrin
BC015822	4.0395761	0.0926	chr3	LTF	Lactotransferrin
NM_002343	4.9773348	0.1161	chr3	LTF	Lactotransferrin
NM_014893	0	0.1291	chrY	NLGN4Y	Neuroligin 4, Y-linked
BC055089	0	0.1412	chr3	CAMP	Cathelicidin antimicrobial peptide
AL133030	4.0395761	0.4397	chr22	KIAA1666	KIAA1666 protein

## References

[1] Anderson LC, Bolling DZ, Schelinski S (2013). Sex differences in the development of brain mechanisms for processing biological motion. *Neuroimage*.

[2] Parks RJ, Howlett SE (2013). Sex differences in mechanisms of cardiac excitation-contraction coupling. *Pflügers Archiv*.

[3] Majek O, Gondos A, Jansen L (2013). Sex differences in colorectal cancer survival: population-based analysis of 164,996 colorectal cancer patients in Germany. *PLoS ONE*.

[4] Kwekel JC, Desai VG, Moland CL, Vijay V, Fuscoe JC (2013). Sex differences in kidney gene expression during the life cycle of F344 rats. *Biology of Sex Differences*.

[5] Donner NC, Lowry CA (2013). Sex differences in anxiety and emotional behavior. *Pflügers Archiv*.

[6] Valentino RJ, van Bockstaele E, Bangasser D (2013). Sex-specific cell signaling: the corticotropin-releasing factor receptor model. *Trends in Pharmacological Sciences*.

[7] McClelland EE, Smith JM (2011). Gender specific differences in the immune response to infection. *Archivum Immunologiae et Therapiae Experimentalis*.

[8] Choudhry MA, Bland KI, Chaudry IH (2007). Trauma and immune response-Effect of gender differences. *Injury*.

[9] Maskew M, Brennan AT, Westreich D (2013). Gender differences in mortality and CD4 count response among virally suppressed HIV-positive patients. *Journal of Women's Health*.

[10] Sankaran-Walters S, Macal M, Grishina I (2013). Sex differences matter in the gut: effect on mucosal immune activation and inflammation. *Biology of Sex Differences*.

[11] Abdullah M, Chai P-S, Chong M-Y (2012). Gender effect on in vitro lymphocyte subset levels of healthy individuals. *Cellular Immunology*.

[12] Scotland RS, Stables MJ, Madalli S, Watson P, Gilroy DW (2011). Sex differences in resident immune cell phenotype underlie more efficient acute inflammatory responses in female mice. *Blood*.

[13] Wikby A, Månsson IA, Johansson B, Strindhall J, Nilsson SE (2008). The immune risk profile is associated with age and gender: findings from three Swedish population studies of individuals 20–100 years of age. *Biogerontology*.

[14] Yan J, Greer JM, Hull R (2010). The effect of ageing on human lymphocyte subsets: comparison of males and females. *Immunity and Ageing*.

[15] Garcia Verdecia B, Saavedra Hernandez D, Lorenzo-Luaces P (2013). Immunosenescence and gender: a study in healthy Cubans. *Immunity & Ageing*.

[16] Straface E, Gambardella L, Brandani M, Malorni W (2012). Sex differences at cellular level ‘cells have a sex’. *Sex and Gender Differences in Pharmacology*.

[17] Giampietri C, Petrungaro S, Filippini A, Ziparo E (2013). Sex-related differences in death control of somatic cells. *Journal of Cellular and Molecular Medicine*.

[18] Korganow A-S, Knapp A-M, Nehme-Schuster H (2010). Peripheral B cell abnormalities in patients with systemic lupus erythematosus in quiescent phase: decreased memory B cells and membrane CD19 expression. *Journal of Autoimmunity*.

[19] Mok MY (2010). The immunological basis of B-cell therapy in systemic lupus erythematosus. *International Journal of Rheumatic Diseases*.

[20] Liossis S-NC, Kovacs B, Dennis G, Kammer GM, Tsokos GC (1996). B cells from patients with systemic lupus erythematosus display abnormal antigen receptor-mediated early signal transduction events. *Journal of Clinical Investigation*.

[21] Reininger L, Winkler TH, Kalberer CP, Jourdan M, Melchers F, Rolink AG (1996). Intrinsic B cell defects in NZB and NZW mice contribute to systemic lupus erythematosus in (NZB x NZW)F1 mice. *Journal of Experimental Medicine*.

[22] Isenberg DA (2006). B cell targeted therapies in autoimmune diseases. *Journal of Rheumatology*.

[23] Garaud J-C, Schickel J-N, Blaison G (2011). B cell signature during inactive systemic lupus is heterogeneous: toward a biological dissection of lupus. *PLoS ONE*.

[24] Becker AM, Dao KH, Han BK (2013). SLE peripheral blood B cell, T cell and myeloid cell transcriptomes display unique profiles and each subset contributes to the interferon signature. *PLoS ONE*.

[25] Sakiani S, Olsen NJ, Kovacs WJ (2013). Gonadal steroids and humoral immunity. *Nature Reviews Endocrinology*.

[26] Oertelt-Prigione S (2012). The influence of sex and gender on the immune response. *Autoimmunity Reviews*.

[27] Candore G, Balistreri CR, Colonna-Romano G (2010). Gender-related immune-inflammatory factors, age-related diseases, and longevity. *Rejuvenation Research*.

[28] Yang L, Hu Y, Hou Y (2006). Effects of 17*β*-estradiol on the maturation, nuclear factor kappa B p65 and functions of murine spleen CD11c-positive dendritic cells. *Molecular Immunology*.

[29] Hao S, Li P, Zhao J, Hu Y, Hou Y (2008). 17*β*-estradiol suppresses cytotoxicity and proliferative capacity of murine splenic NK1.1+ cells. *Cellular and Molecular Immunology*.

[30] Xie H, Hua C, Sun L (2011). 17*β*-estradiol induces CD40 expression in dendritic cells via MAPK signaling pathways in a minichromosome maintenance protein 6-dependent manner. *Arthritis and Rheumatism*.

[31] Xu Y, Fan H, Li X, Sun L, Hou Y (2012). beta-Estradiol enhances response of mice spleen B cells elicited by TLR9 agonist. *Cellular Immunology*.

[32] Lee T-P, Chiang B-L (2012). Sex differences in spontaneous versus induced animal models of autoimmunity. *Autoimmunity Reviews*.

[33] Hochberg MC (1997). Updating the American College of Rheumatology revised criteria for the classification of systemic lupus erythematosus. *Arthritis and Rheumatism*.

[34] Bombardier C, Gladman DD, Urowitz MB, Caron D, Chi Hsing Chang CHC (1992). Derivation of the SLEDAI: a disease activity index for lupus patients. *Arthritis and Rheumatism*.

[35] Actor JK, Hwang S-A, Kruzel ML (2009). Lactoferrin as a natural immune modulator. *Current Pharmaceutical Design*.

[36] Kin NW, Chen Y, Stefanov EK, Gallo RL, Kearney JF (2011). Cathelin-related antimicrobial peptide differentially regulates T- and B-cell function. *European Journal of Immunology*.

[37] Zilbauer M, Jenke A, Wenzel G (2011). Intestinal alpha-defensin expression in pediatric inflammatory bowel disease. *Inflammatory Bowel Diseases*.

[38] Hewagama A, Patel D, Yarlagadda S, Strickland FM, Richardson BC (2009). Stronger inflammatory/cytotoxic T-cell response in women identified by microarray analysis. *Genes and Immunity*.

[39] Aravamudan B, VanOosten SK, Meuchel LW (2012). Caveolin-1 knockout mice exhibit airway hyperreactivity. *American Journal of Physiology—Lung Cellular and Molecular Physiology*.

[40] Guo C-J, Yang X-B, Wu Y-Y (2011). Involvement of caveolin-1 in the Jak-Stat signaling pathway and infectious spleen and kidney necrosis virus infection in mandarin fish (Siniperca chuatsi). *Molecular Immunology*.

[41] Gilling CE, Mittal AK, Chaturvedi NK, Iqbal J, Aoun P (2012). Lymph node-induced immune tolerance in chronic lymphocytic leukaemia: a role for caveolin-1. *British Journal of Haematology*.

[42] Caserta S, Nausch N, Sawtell A (2012). Chronic infection drives expression of the inhibitory receptor CD200R, and its ligand CD200, by mouse and human CD4 T cells. *PLoS ONE*.

[43] Uyama T, Jin X-H, Tsuboi K, Tonai T, Ueda N (2009). Characterization of the human tumor suppressors TIG3 and HRASLS2 as phospholipid-metabolizing enzymes. *Biochimica et Biophysica Acta*.

[44] Schmidt J, Riechers A, Bosserhoff AK (2013). MIA–a new target protein for malignant melanoma therapy. *Histology and Histopathology*.

[45] Hara-Chikuma M, Chikuma S, Sugiyama Y (2012). Chemokine-dependent T cell migration requires aquaporin-3-mediated hydrogen peroxide uptake. *The Journal of Experimental Medicine*.

[46] Bund D, Buhmann R, Gokmen F, Zorn J, Kolb HJ (2013). Minor histocompatibility antigen UTY as target for graft-versus-leukemia and graft-versus-haematopoiesis in the canine model. *Scandinavian Journal of Immunology*.

[47] Gil J, Busto EM, Garcillan B, Chean C (2011). A leaky mutation in CD3D differentially affects alphabeta and gammadelta T cells and leads to a Talphabeta-Tgammadelta+B+NK+ human SCID. *Journal of Clinical Investigation*.

[48] Sud N, Wiseman DA, Black SM (2010). Caveolin 1 is required for the activation of endothelial nitric oxide synthase in response to 17*β*-estradiol. *Molecular Endocrinology*.

[49] Li Y, Zhao LD, Tong LS (2012). Aberrant CD200/CD200R1 expression and function in systemic lupus erythematosus contributes to abnormal T-cell responsiveness and dendritic cell activity. *Arthritis Research & Therapy*.

[50] Liu Y, Hong X, Kappler J (2003). Ligand-receptor binding revealed by the TNF family member TALL-1. *Nature*.

[51] Lacotte S, Decossas M, le Coz C (2013). Early differentiated CD138(high) MHCII+ IgG+ plasma cells express CXCR3 and localize into inflamed kidneys of lupus mice. *PLoS ONE*.

[52] Tian Y, Stamova B, Jickling GC (2012). Y chromosome gene expression in the blood of male patients with ischemic stroke compared with male controls. *Gender Medicine*.

[53] Koenecke C, Förster R (2009). CCR9 and inflammatory bowel disease. *Expert Opinion on Therapeutic Targets*.

[54] Liu ZK, Wang RC, Han BC, Yang Y, Peng JP (2012). A novel role of IGFBP7 in mouse uterus: regulating uterine receptivity through Th1/Th2 lymphocyte balance and decidualization. *PLoS ONE*.

[55] Aporta A, Catalan E, Galan-Malo P (2013). Granulysin induces apoptotic cell death and cleavage of the autophagy regulator Atg5 in human hematological tumors. *Biochemical Pharmacology*.

[56] Ewen CL, Kane KP, Bleackley RC (2013). Granzyme H induces cell death primarily via a Bcl-2-sensitive mitochondrial cell death pathway that does not require direct Bid activation. *Molecular Immunology*.

[57] de Weerd NA, Vivian JP, Nguyen TK (2013). Structural basis of a unique interferon-beta signaling axis mediated via the receptor IFNAR1. *Nature Immunology*.

[58] Nickerson KM, Cullen JL, Kashgarian M, Shlomchik MJ (2013). Exacerbated autoimmunity in the absence of TLR9 in MRL.Fas(lpr) mice depends on Ifnar1. *The Journal of Immunology*.

[59] Lobo EO, Zhang Z, Shively JE (2009). Pivotal Advance: CEACAM1 is a negative coreceptor for the B cell receptor and promotes CD19-mediated adhesion of B cells in a PI3K-dependent manner. *Journal of Leukocyte Biology*.

[60] Busslinger M (2004). Transcriptional control of early B cell development. *Annual Review of Immunology*.

[61] DeKoter RP, Schweitzer BL, Kamath MB (2007). Regulation of the interleukin-7 receptor *α* promoter by the Ets transcription factors PU.1 and GA-binding protein in developing B cells. *Journal of Biological Chemistry*.

[62] Pogue SL, Preston BT, Stalder J, Bebbington CR, Cardarelli PM (2004). The receptor for type I IFNs is highly expressed on peripheral blood B cells and monocytes and mediates a distinct profile of differentiation and activation of these cells. *Journal of Interferon and Cytokine Research*.

[63] Sullivan NL, Eickhoff CS, Zhang X, Giddings OK, Lane TE, Hoft DF (2011). Importance of the CCR5-CCL5 axis for mucosal Trypanosoma cruzi protection and B cell activation. *Journal of Immunology*.

[64] Serre K, Cunningham AF, Coughlan RE (2012). CD8 T cells induce T-bet-dependent migration toward CXCR3 ligands by differentiated B cells produced during responses to alum-protein vaccines. *Blood*.

[65] Xu Y, Xu Q, Yang L (2013). Gene expression analysis of peripheral blood cells reveals Toll-like receptor pathway deregulation in colorectal cancer. *PLoS One*.

[66] Menon R, di Dario M, Cordiglieri C (2012). Gender-based blood transcriptomes and interactomes in multiple sclerosis: involvement of SP1 dependent gene transcription. *Journal of Autoimmunity*.

[67] Choi KY, Chow LN, Mookherjee N (2012). Cationic host defence peptides: multifaceted role in immune modulation and inflammation. *Journal of Innate Immunity*.

[68] Steinstraesser L, Kraneburg U, Jacobsen F, Al-Benna S (2011). Host defense peptides and their antimicrobial-immunomodulatory duality. *Immunobiology*.

[69] Nadkarni S, McArthur S (2013). Oestrogen and immunomodulation: new mechanisms that impact on peripheral and central immunity. *Current Opinion in Pharmacology*.

