# Supplementary material for: Gender Differences of B Cell Signature in Healthy Subjects Underlie Disparities in Incidence and Course of SLE Related to Estrogen
Source: J Immunol Res. 2014 Feb 13;2014:814598. doi: 10.1155/2014/814598 (PMC3987971; doi:10.1155/2014/814598)

Suppl. Table 1. 26 down-regulated expression genes in SLE females vs SLE males, which of most genes were linked to Y chromosome

Gene ID	q-value(%)	Fold Change	Chromosomal	Symbol	Full name
NM_004653	0	0.0011	chrY	SMCY	Smcy homolog, Y-linked (mouse)
D87072	0	0.0019	chrY	SMCY	Smcy homolog, Y-linked (mouse)
NM_032576	0	0.0021	chrY	CYorf15B	chromosome Y open reading frame 15B
NM_004654	0	0.0027	chrY	USP9Y	ubiquitin specific peptidase 9, Y-linked (fat facets-like, Drosophila)
NM_004681	0	0.0028	chrY	EIF1AY	eukaryotic translation initiation factor 1A, Y-linked
BC100905	0	0.0041	chrY	RPS4Y1	ribosomal protein S4, Y-linked 1
NM_004660	0	0.0055	chrY	DDX3Y	DEAD (Asp-Glu-Ala-Asp) box polypeptide 3, Y-linked
NM_001008	0	0.0072	chrY	RPS4Y1	ribosomal protein S4, Y-linked 1
NM_182659	0	0.0087	chrY	UTY	ubiquitously transcribed tetratricopeptide repeat gene, Y-linked
AF332239	0	0.0096	chrY	TTTY10	testis-specific transcript, Y-linked 10
**NM_001005852**	**0**	**0.0102**	**chrY**	**CYorf15A**	**chromosome Y open reading frame 15A**
BC035312	0	0.0103	chrY	CYorf15B	chromosome Y open reading frame 15B
BC110817	0	0.0104	chrY	CYorf15A	chromosome Y open reading frame 15A
NM_003411	0	0.0113	chrY	ZFY	zinc finger protein, Y-linked
NM_182660	0	0.0221	chrY	UTY	ubiquitously transcribed tetratricopeptide repeat gene, Y-linked
NM_007125	0	0.0285	chrY	UTY	ubiquitously transcribed tetratricopeptide repeat gene, Y-linked
BC034942	0	0.0572	chrY	DDX3Y	DEAD (Asp-Glu-Ala-Asp) box polypeptide 3, Y-linked
**BC093959**	**4.9773348**	**0.0635**	**chr8**	**DEFA4**	**defensin, alpha 4, corticostatin**
**BC015823**	**0**	**0.0849**	**chr3**	**LTF**	**lactotransferrin**
**BC015822**	**4.0395761**	**0.0926**	**chr3**	**LTF**	**lactotransferrin**
NM_001039567	0	0.111	chrY	RPS4Y2	ribosomal protein S4, Y-linked 2
**NM_002343**	**4.9773348**	**0.1161**	**chr3**	**LTF**	**lactotransferrin**
**NM_014893**	**0**	**0.1291**	**chrY**	**NLGN4Y**	**neuroligin 4, Y-linked**
**BC055089**	**0**	**0.1412**	**chr3**	**CAMP**	**cathelicidin antimicrobial peptide**
BC005248	0	0.2904	chrY	EIF1AY	eukaryotic translation initiation factor 1A, Y-linked
**AL133030**	**4.0395761**	**0.4397**	**chr22**	**KIAA1666**	**KIAA1666 protein**

*Bold words showed eight DEGs have non-significant different between healthy females and healthy males.

Suppl. Table 2. Enriched signaling pathways with significant association with 26 DEGs (SLE females/ SLE males)

	**No. of genes**	p-Value
Kegg pathways		
Ribosome	1	0.019865
Genmapp pathways		
GO_Samples--Biological process--spermatogenesis	2	2.42E-04
GO_Samples--Cellular component--male gamete generation	2	2.42E-04
GO_Samples--Cellular component--gametogenesis	2	3.68E-04
Tissue-specific--Hs_1-Tissue-Blood_and_Lymph	2	4.30E-04
GO_Samples--Biological process--sexual reproduction	2	5.67E-04
GO_Samples--Biological process--reproduction	2	9.37E-04
Contributed--cellular_process--Hs_Translation_Factors	1	0.009782
Contributed--molecular_function--Hs_Ribosomal_Proteins	1	0.01716
GO_Samples--Molecular function--translation factor activity, nucleic acid binding	1	0.019286
GO_Samples--Biological process--cell ion homeostasis	1	0.019286
GO_Samples--Biological process--di-, tri-valent inorganic cation transport	1	0.019479
GO_Samples--Molecular function--translation regulator activity	1	0.019672
GO_Samples--Molecular function--cysteine-type endopeptidase activity	1	0.020251
GO_Samples--Biological process--ion homeostasis	1	0.021216
GO_Samples--Biological process--cell homeostasis	1	0.021408
GO_Samples--Biological process--ubiquitin-dependent protein catabolism	1	0.021986
GO_Samples--Molecular function--cysteine-type peptidase activity	1	0.024104
GO_Samples--Molecular function--helicase activity	1	0.024872
GO_Samples--Molecular function--serine-type endopeptidase activity	1	0.028517
GO_Samples--Biological process--humoral immune response	1	0.030048
GO_Samples--Molecular Function--catalytic activity	1	0.031386
GO_Samples--Biological process--translation	1	0.03215
GO_Samples--Molecular function--lipid binding	1	0.035961
GO_Samples--Biological process--response to chemical substance	1	0.039189
GO_Samples--Biological process--ubiquitin cycle	1	0.046371
GO_Samples--Cellular component--cytosol	1	0.048818
GO_Samples--Cellular component--extracellular space	1	0.052195

Suppl. Table 3. Top ten enriched signaling pathways with significant association with DEGs (SLE females/SLE males)

	No. of genes	p-Value
**KEGG pathways**		
Ribosome	1	0.019865
**Biocarta pathways**		
novel		
**Genmapp pathways**		
spermatogenesis	2	2.42E-04
male gamete generation	2	2.42E-04
gametogenesis	2	3.68E-04
Blood_and_Lymph	2	4.30E-04
sexual reproduction	2	5.67E-04
reproduction	2	9.37E-04
Translation_Factors	1	0.009782
Ribosomal_Proteins	1	0.01716
translation factor activity, nucleic acid binding	1	0.019286
cell ion homeostasis	1	0.019286

Suppl. Table 4. Top ten enriched signaling pathways with significant association with estrogen-relevant DEGs (SLE females/SLE males)

	No. of genes	p-Value
**KEGG pathways**		
Ribosome	1	0.013287258
**Biocarta pathways**		
novel		
**Genmapp pathways**		
Blood_and_Lymph	2	1.80E-04
Translation_Factors	1	0.006531869
Ribosomal_Proteins	1	0.011472277
translation factor activity, nucleic acid binding	1	0.012898568
cell ion homeostasis	1	0.012898568
di-, tri-valent inorganic cation transport	1	0.013028145
translation regulator activity	1	0.013157709
ion homeostasis	1	0.014193707
cell homeostasis	1	0.014323143
helicase activity	1	0.016650571

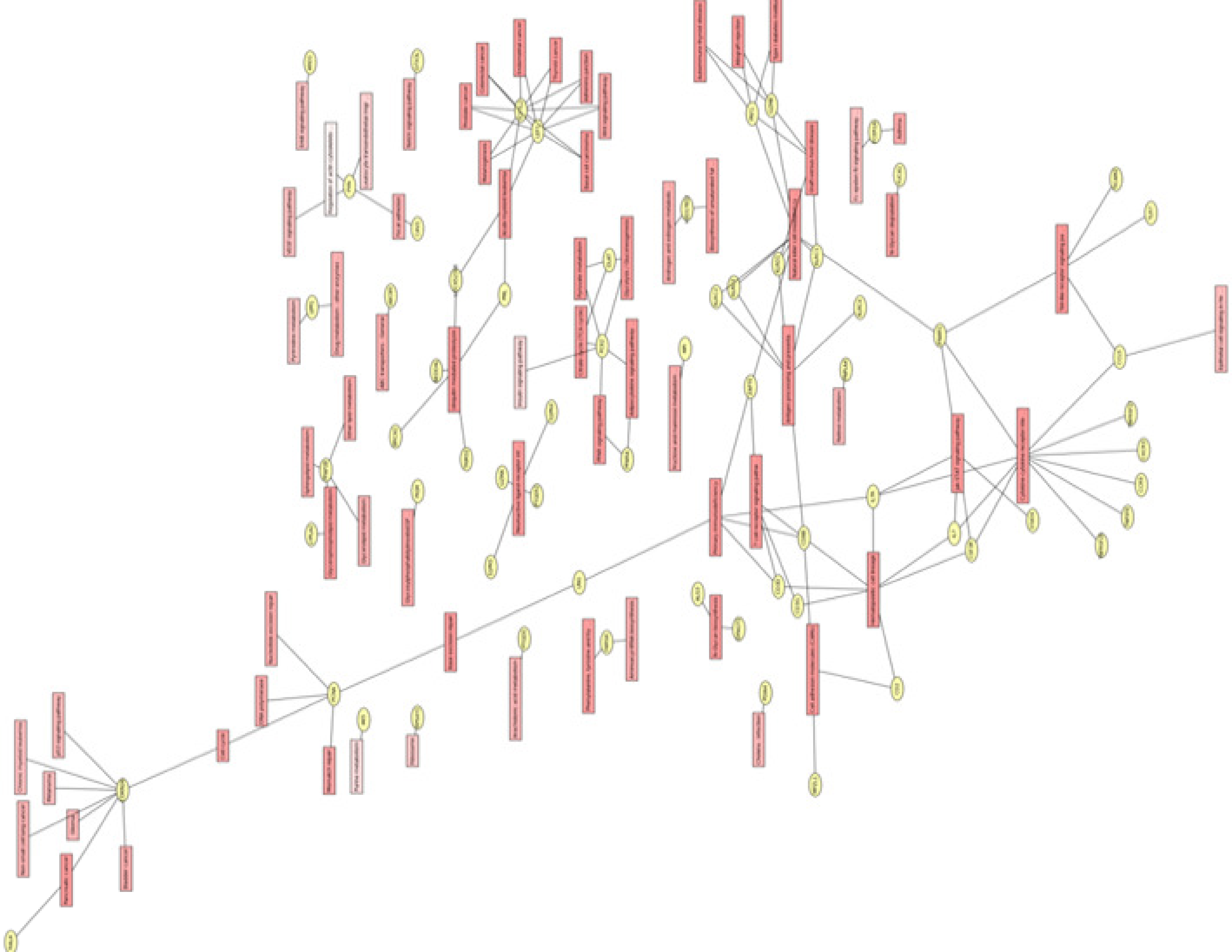

Supplement: Supplementary file 1 — The differential gene expressions (DEGs) in B cells of SLE males and females were determined by microarray. Only 26 gene expressions were down-regulated in SLE females versus SLE males, which of most genes were linked to Y chromosome (Supplementary Table 1). And eight DEGs (CYorf15A, DEFA4, NLGN4Y, CAMP, KIAA1666 and three LTF varients) have non-significant different between healthy females and healthy males (Supplementary Table 1). The KEGG pathways analysis revealed that pathways associated with B cells of SLE males were Ribosome-relevant signaling pathways, indicating the abundant protein synthsis in abnormal B cells (Supplementary Table 2). The Genmapp pathways analysis 27 enriched signaling pathways, including biological process spermatogenesis, cellular component male gamete generation, and cellular component gametogenesis, et al (Supplementary Table 2). Top ten enriched signaling pathways with significant association with DEGs or with estrogen-relevant DEGs (SLE females/SLE males) were provided in Supplementary Table 3 and Supplementary Table 4. 358 DEGs in B cells of healthy females' and males' comparison were subjected to perform the signaling pathway analysis based on KEGG databases. A group of 65 DEGs are found connected functionally, including the estrogen-relevant DEGs described in Figure 4 (Figure S1). [file 814598.f1.pdf]
